# Retraction for Nazir et al., “Characterization, taxonomic classification, and genomic analysis of two newly isolated bacteriophages with potential to infect *Escherichia coli*”

**DOI:** 10.1128/spectrum.02230-23

**Published:** 2025-06-24

**Authors:** Amina Nazir, Lulu Li, Fei Li, Yigang Tong, Yuqing Liu, Yibao Chen

## RETRACTION

e02230-23, 2024, https://doi.org/10.1128/spectrum.02230-23. Following the publication of our paper, we continued to delve deeper into our research and reacquired transmission electron microscope (TEM) images in Fig. 1a. Upon careful comparison and analysis, we observed discrepancies between the newly acquired images and those previously submitted. This discovery led us to realize that the TEM images in Fig. 1a may not accurately represent the true experimental results, potentially affecting readers’ understanding and judgment of our research conclusions.

Fully aware of the importance of academic integrity and rigor, we have decided to retract the paper to avoid misleading the academic community. We deeply regret this incident and extend our sincere apologies to the journal and all readers.

