# Expression of Concern for Nazir et al., “Characterization, taxonomic classification, and genomic analysis of two newly isolated bacteriophages with potential to infect *Escherichia coli*”

**DOI:** 10.1128/spectrum.02252-24

**Published:** 2024-11-18

**Authors:** American Society for Microbiology

## EXPRESSION OF CONCERN

The American Society for Microbiology (ASM) and *Microbiology Spectrum* are issuing this Expression of Concern regarding the following publication:

Nazir A, Li L, Li F, Tong Y, Liu Y, Chen Y. 2024. Characterization, taxonomic classification, and genomic analysis of two newly isolated bacteriophages with potential to infect *Escherichia coli*. Microbiol Spectr e02230-23. https://doi.org/10.1128/spectrum.02230-23.

*Microbiology Spectrum* was notified by a concerned reader about discrepancies in the data presented in Fig. 1a, which shows a micrograph of phage IME177 obtained using a transmission electron microscope. Furthermore, concerns were raised about the discordance between the genomics data and the results shown in Fig. 1a.

ASM is independently verifying these concerns and has initiated an inquiry with the authors. In publishing this Expression of Concern, ASM is following its Publishing Ethics Policies and Procedures and, as a member of the Committee on Publication Ethics (COPE), follows its guidelines in this regard. ASM and *Microbiology Spectrum* have provided a deadline for the corresponding authors to provide us with an explanation. This Expression of Concern will be updated accordingly thereafter.

