# Supplementary material for: Retraction for Nazir et al., “Characterization, taxonomic classification, and genomic analysis of two newly isolated bacteriophages with potential to infect Escherichia coli”
Source: Microbiol Spectr. 2025 Jun 24;13(8):e02230-23. doi: 10.1128/spectrum.02230-23 (PMC12443368; doi:10.1128/spectrum.02230-23)
Supplement: Related texts

# Research article: Retracted: Characterization, taxonomic classification, and genomic analysis of two newly isolated bacteriophages with potential to infect *Escherichia coli*

**DOI:** 10.1128/spectrum.02230-23

**Authors:** Amina Nazir, Lulu Li, Fei Li, Yigang Tong, Yuqing Liu, Yibao Chen

**Affiliations:** 1 Shandong Key Laboratory of Animal Disease Control and Breeding, Institute of Animal Science and Veterinary Medicine, Shandong Academy of Agricultural Sciences, Jinan, Shandong, China; 2 Key Laboratory of Livestock and Poultry Multi-omics of MARA, China-UK Joint Laboratory of Bacteriophage Engineering, Jinan, China; 3 College of Life Science and Technology, Beijing University of Chemical Technology, Beijing, China; The University of Tennessee Knoxville, Knoxville, Tennessee, USA

**Keywords:** *Escherichia coli*, bacteriophage, antibiotics, kayfunavirus genus, taxonomy and genomic

## Abstract

*Escherichia coli* is a pathogenic bacterium that is widely distributed and can lead to serious illnesses in both humans and animals. As there is rising incidence of multidrug resistance among these bacteria, it has become imperative to discover alternative therapies beyond antibiotics to effectively treat such infections. Bacteriophage (phage) therapy has the potential to treat infections caused by *E. coli*, as phages contain enzymes that can cause lysis or destruction of bacterial cells. Simultaneously, the easy accessibility and cost-effectiveness of next-generation sequencing technologies have led to the accumulation of a vast amount of phage sequence data. Here, phages IME177 and IME267 were isolated from sewage water of a hospital in China. Modern phylogenetic approaches and key findings from the genomic analysis revealed that phages IME177 and IME267 are classified as members of the *Kayfunavirus* genus, *Autographiviridae* family, and a newly proposed *Suseptimavirus* genus under subfamily *Gordonclarkvirinae*, respectively. Further, the Kuravirus genus reshaped into three different genera: *Kuravirus*, *Nieuwekanaalvirus*, and *Suspeptimavirus*, which are classified together under a higher taxonomic rank (subfamily) named *Gordonclarkvirinae*. No genes related to virulence were detected in the genomes of the phages IME177 and IME267. Both phages exhibited a high degree of resilience to a wide range of conditions, including pH, temperature, exposure to chloroform, and UV radiation. Phages IME177 and IME267 are promising biological agents that can infect *E. coli*, making them suitable candidates for use in phage therapies.

**IMPORTANCE:**

Biological and taxonomic characterization of phages is essential for facilitating the development of effective strategies for phage therapy and disease control. *Escherichia coli* phages are incredibly diverse, and their isolation and classification help us understand the scope and nature of this diversity. By identifying new phages and grouping them into families, we can better understand the genetic and structural variations between phages and how they affect their infectivity and interactions with bacteria. Overall, the isolation and classification of *E. coli* phages have broad implications for both basic and applied research, clinical practice, and public health.
